# Description of Two Cases of Tetanus

**Published:** 1817-01

**Authors:** 


*For the London Medical and Physical Journal.*

### *Description of Two Cases of Tetanus;* by Dr. Cross.

J. MURDOCH, apprentice to Mr. J. Caldwell, flesher, Main-street, Gorbals, on Oct. 21, 1815, consulted Mr. Peter Miller, surgeon, Hutcheson Town, about a stiffness of the jaws. He was then 13 years of age, four feet nine inches high, stout, well-shaped, and of a fair complexion. Mr. Caldwell, in whose neighbourhood the boy had been brought up, took a fancy for him from his childhood, on account of his uncommon alacrity and obliging disposition; so that, although his parents were poor, he has for the most part lived with his present master, and enjoyed generous diet. He could assign no cause for the stiffness of his jaws; but at length on his left leg a sore was discovered, which led back to the 9th instant, when, at the building of a hay-stack, he received from the prong of a hay fork, thrown at him "in fun," a small wound four inches above the left inner ankle, and about a line or two behind the edge of the tibia. Although the wound was pretty deep, yet no blood issued, except a dot "about the size of the head of a pin." The place soon became swelled and red, but not very painful until the 12th, when he made an excursion with some boys to a glen, five miles distant, to gather bramble-berries, got himself drenched with rain, and after his return neglected to change his clothes. As the sore had become painful, and its edges

 hard, hard, Mrs. Caldwell applied a poultice. Hitherto the matter discharged had been of proper consistence, but now became more and more watery, while the pain became less and less. On the morning of the 18th, he complained of a difficulty in chewing a piece of loaf-bread. In the evening he could not open his mouth to the usual extent, and felt considerable difficulty in chewing. On the morning of the 19th, in supping his porridge, he could scarcely get the spoon into his mouth. In the forenoon of the 20th he had wheeled a cow's hide to a tan-work a quarter of a mile distant, and was looking into a tan-hole, when he became giddy, and nearly dropped in. On getting home with his barrow, he had to go to bed, and in a few hours became free of giddiness, but the stiffness of the jaws was slowly gaining ground. On the 21st, in the forenoon, his arm, in throwing a stone at a dog, fell powerless by his side, and the stone dropped from his hand. He does not know whether the arm became relaxed or stiff. Till the evening of the 21st, the stiffness was limited to the jaws. Now the tongue also became sore and stiff, as if it had not sufficient room. Having become alarmed at the increase of symptoms, he now applied, as formerly mentioned, to Mr. Miller, who immediately stripped him, and discovered on the left leg the sore slightly inflamed, but devoid of pain, and discharging a watery fluid. ℈iſs. of compound jalap was prescribed, and he was desired to return next morning.

22. Morning.—Had slept little; physic had operated; stiffness had extended to the back of the neck. The proposal of sending him to the infirmary alarmed him, so that he hurried home, but almost fell two or three times on the road. In the afternoon, being unable to walk to Mr. Miller, he was visited about two o'clock. The sore was enlarged, and pencilled with caustic: ℈i. of ipecac. and gr. $\frac{1}{4}$ of tart. antim. were given as an emetic, and operated slightly. What came up, he says, was very sour. He was now put into the warm bath, after which the following mixture was ordered:—℞. Tinct. opii, gutt. lxxx. eth. sulph. ʒſs. aq. cinnam. ℥i. mix. the half to be taken immediately, and the remainder in half an hour. From this time forward he was closely attended by Mr. Miller, and occasionally by his partner, Mr. M'Lean.

Eleven at night.—A paroxysm had thrown him backwards to the floor. A table spoonful of the following mixture, ℞. Tinct. opii, ℥ii. sp. eth. nitr. ʒiſs. aq. cinnam. ℥iii. mix. was ordered to be swallowed every second hour. Camphorated mercurial ointment was to be rubbed occasionally over his spine, and a dose of cal. jal. and ginger was given.

On

On the 23d, doses of calomel and opium were ordered. The ointment, the warm bath, and the purgative, were continued; and in the evening an injection was administered; still the symptoms continued to increase.

On the 24th, the cold bath, in place of the warm, was ordered. With a little force, he could be bent to the sitting posture in the bath. The former prescriptions were continued. Still the symptoms were increasing.

On the 25th, he could not be made to sit in the cold bath. A solution of ℥iſs. of sulph. magn. in cinnamon water was ordered.

26th.—The notes do not mention whether the physic operated. The cold affusion was ordered. Opiate frictions were set a-going, and a dose of jal. coloc. and ginger was given.

On the 27th, I was requested to attend, and saw him, for the first time, at twelve o'clock at night. By this time his jaws were closely and immoveably locked; and his whole body was stiff, and bent backwards into an arch, so that he rested on the occiput and heels. From the first he enjoyed but little sleep; now it had entirely departed from him. His urine had been all along scanty. The skin was dry, except over the head, face, and neck, which were for the most part bedewed with sweat. His chief pain lay in the pit of the stomach, where there was an evident swelling. He described the pain to proceed from this spot over the body. For the last twenty-eight hours purgatives and injections had failed to procure any alvine discharge. Deglutition had all along been difficult; but now he could scarcely manage to swallow any fluid introduced between his teeth, and never without inducing a paroxysm. The paroxysms were now frequent, and accompanied with difficulty of breathing, sometimes almost amounting to suffocation, and with palpitation of the heart, which could be seen and heard. His breath had the mercurial fœtor. As the medicines were now producing no effect; as the disease seemed to be fast approaching to a fatal termination, for which indeed the poor boy became impatiently desirous, and was calling upon his nurse to put an end to his miserable existence; and as hydrophobia, which is very much a kin to tetanus, has been of late repeatedly cured by blood-letting, we resolved to give the lancet a fair trial. Accordingly we drew from the arm ℥xvi. of blood. Before the blood ceased to flow, he was able to open his jaws to the extent of half an inch. The opportunity was seized of giving a smart dose of purgative medicine, which was ordered to be followed by repeated doses of castor oil. An injection, which was thrown into the the rectum by a syringe, was immediately ejected past the syringe with considerable violence against the foot of the bed. The relaxation produced by the bleeding gradually disappeared, and in a few hours the jaws became as locked, and the body as stiff, as ever. The bleeding was repeated in about ten hours afterwards, and produced a relaxation of the jaws, though not to the former extent. The physic had now begun to operate. A smart dose of calomel was given, and ordered to be followed by a solution of sulph. magn. in cinnamon water. Repeated doses of sp. eth. nitr. were also ordered. In a few hours the effects of the bleeding had almost disappeared. On the evening of the 28th, and on the 29th, the bleeding was repeated, and on both occasions produced considerable relaxation, though not to the first amount. He had now been bled four times, and had lost 5 ℔ of blood. After every bleeding the pulse became exceedingly quick; but by and by subsided to about 110. Brisk purging with sulph. magn. and an occasional dose of cal. was kept up. Under all this depletion, his whole food was a little soup, so that he had now become exceedingly weak. For fear he would die in our hands, we did not venture to take more blood till the 30th, on the morning of which day he seemed a little recruited. The body still formed an arch from occiput to heel; and the jaws were locked, though not quite so immoveably, as before the bleedings. This difference, however, we were disposed to place to the account of debility. From the regular temporary amelioration of the symptoms after every bleeding; from the inefficacy of every thing else; and from the hopelessness of the case, if left to itself; we resolved, after much hesitation, to make one desperate trial more with the lancet. The vein was opened. A considerable stream of blood issued, and the finger was held to the pulse till it became exceedingly quick and feeble; when the poor boy all at once became exceedingly low, and seemed ready to expire at every paroxysm. Having tied up the arm, we retired hastily to another room, and agreed to let him have as much wine as the friends could get him to swallow, more as a mere cordial, than as a medicine from which any benefit could now be expected, and announced that we did not expect that the patient would survive long. Next morning, however, we found our young patient in a state of intoxication, and much relieved. As there had been all along a scarcity of urine, we substituted gin to-day for the wine, and kept him in a state of half intoxication for ten days. Purgation was at the same time kept up. During this period it was remarked that he ejected his urine to a great distance; on one occasion to the distance of fourteen feet. His recovery was very slow and gradual; so that, for many days after the gin was commenced, he was lifted about like a piece of wood. Not long after he began to walk about, his motions were stiff and slow. He has, however, long since regained his former health, and strength, and activity.

---

James Campbell Wright, Cowcaddens, aged 49, five feet and seven inches high; thick, muscular, well-shaped, strong, and agile; the hair on the face red; the hair of the head formerly lightish brown, but, since the late shavings and blisterings, approaching to black; has all along enjoyed excellent health; has occasionally taken a hearty glass with his friends, but upon the whole has been a man of sober and religious habits. On January 27, 1816, at eight o'clock in the morning, in lifting a deal, a little splinter, not so thick as an ordinary pin, made its way into the middle of the fore-part of the first phalanx of the right thumb, and was immediately taken out by his comrade. The circumstance was quite forgotten till next day, (Sunday,) about twelve o'clock midday, when the part became painful and swollen. About five o'clock in the afternoon, a small suppuration of about the size of a pin's head was discovered in the spot from which the splinter had been extracted. As soon as the matter, which was of thick consistence, was let out, the pain diminished, but the thumb continued to increase gradually in size, and to become weaker and weaker. By ten o'clock next morning the whole right hand had become so powerless, that he was unable to lift a tool, or even a breakfast-knife. By twelve o'clock, the hand had become cold, and he was warming it at the fire, when in an instant he was seized with an excruciating pain in the situation of the carpal bones; the back of the head forthwith swelled, and became a little red. At three o'clock he applied to Mr. Roderick Gray, surgeon, Cowcaddens, who ordered the whole hand to be covered with cloths dipped in vinegar and strong spirits. No relief was obtained. As the pain was increasing, and as Mr. Gray was from home, I was called in the evening. He was screaming aloud, and moving restlessly from place to place in severe agony. There was a slight ruffling of the cuticle, where the speck of suppuration had formed. The chief pain was in the first phalanx of the thumb. I immediately made an incision down to the bone from the borders of the first joint to the tip. A good deal of black blood issued, but no pus could be discerned. The whole hand was immediately immersed in warm water, and afterwards covered with a poultice, and a smart dose of cal.

and and jal. was ordered. The pain of the thumb was immediately removed, and the pain in the back of the hand much mitigated.

On the morning of the 30th, I met Mr. Gray in consultation. We learned that since the incision was made into the thumb, it had been free of pain, but that in about half an hour the pain in the back of the hand gradually increased, and at length produced screaming and restlessness, as before. We applied eight leeches, and ordered them to be followed by warm fomentations, and afterwards by a large poultice. A smart dose of calomel and jalap was given in the evening.

On the 21st, the same number of leeches was applied, and purgation kept up. Still the pain and swelling increased.

On February 1, eight leeches more were applied. While they were at work, he felt a pain suddenly ascend from the hand to the back part of the head. The hand now became easier, but the pain in the back part of the head became proportionally violent. The head was shaved, and rubifaciants, and sinapisms, and blisters, were successively applied, with some temporary, but no permanent, benefit.

By the evening of the 4th, the neck and jaws had become somewhat stiff and painful, and uncommon anxiety was expressed in the face. Mr. Gray having shortly before had a case of bruise which fell a victim to tetanus, and being struck with the similarity of the symptoms before him with those which had so lately made a deep impression on his mind, was the first to discern the approach of this horrific disease. On examining more attentively, we found that the jaws could not be fully opened, and that the stiffness of the neck was considerable. As, however, calomel had been repeatedly given, and might possibly be the cause of all this pain and stiffness, we were willing to wait till morning. An ounce of castor oil was in the mean time given.

Mr. Gray saw him at seven next morning, and found tetanus advancing with rapid strides. At ten we met. By this time the jaws during the intermissions scarcely admitted the point of the little finger, and during the paroxysms were closely locked. The stiffness of the neck was greatly increased. The head was fixed immoveably during the intermissions; and during the paroxysms was drawn forward, with the face turned a little towards the left, and with the occiput of course inclined as far towards the right. Often while the attendants were laying him back in bed, and when the head was yet a few inches from the pillow, he was drawn forward with a sudden convulsive jerk to the sitting posture. There was always great difficulty of deglutition, and, during the paroxysms, of respiration. We immediately filled nine

 large large sized tea-cups with blood from his arm. The quantity, taking in what was in the plate and on the floor, must have exceeded four pounds; and the stream was the largest that either of us ever witnessed. The tetanic symptoms immediately abated. A few pil. aloes c. coloc. were given, and gr. iiiſs. of opium were left to be taken in an hour.

In the evening the jaws and neck were still a little stiff, and there was considerable pain in the back part of the head. We contented ourselves with giving a little physic.

Next morning, at ten o'clock, finding not only the stiffness of the jaws and neck, but also the pain in the back of the head, greatly increased, we filled five saucers with blood from the temporal artery. The quantity of blood might amount to ℥xxv. During the operation he almost fainted twice. The same dose as yesterday of opium, preceded by a few purgative pills, was ordered. From this time forward we did not witness one formidable symptom of the disease, although for eight weeks he continued to be more or less troubled with convulsive contortions of the head. Purgatives during the day, and opiates at night, were continued till every spasmodic symptom disappeared. To follow out the history of the hand is at present unnecessary. Suffice it to mention, that at this moment a probe can be made to rattle amongst the carpal bones, and that he is hesitating about allowing an operation to be performed. In other respects he is at present in perfect health and strength.

---

The subserviency and adaptation of the voluntary powers throughout all the animated world to the cravings of the stomach, and the intimate sympathy that is conspicuous at every step of pathological research between the stomach and the voluntary organs, warrant the conjecture that the stomach is the primary seat of this disease. The pain and swelling in the epigastric region, and the appearances after death, almost confirm this opinion. I attended a farmer a few years ago in the parish of Cambernauld, who laboured under an hysterical disease, which passed through the following succession of symptoms every morning:—He became languid, and unable to speak; the head by and by fell down upon the breast; then, with both fists clenched, he gave the breast a number of most unmerciful blows; then with gaping mouth bellowed out a wild convulsive laugh; then served the head as he had done the chest; then screamed out another wild laugh; after which he often got free belching off flatus from the stomach, and soon recovered. But when the flatus could not at this period escape, his whole body became stiff and straight as the trunk of a tree, and rolled along the floor; at length, after another laugh, and free belching, the fit terminated. I call it an hysterical disease, in distinction to epileptic, because he could hear and remember what was said to him during the fit. In this case the stools, which were regularly preserved for my inspection, emitted large bubbles of gas. This man, before the fits had entirely left him, underwent, by advice of a strolling doctress, the following notable remedy:—A bannock made of his urine and meat was given to the dog. Soon after this the fits entirely disappeared. The cure was ascribed to the doctress; and the medicines, in spite of every remonstrance, were discontinued. The disease has, it seems, lately returned. From this case, I argue that, if the œsophagus had persevered in refusing exit to the flatus, the fit would have continued, and the man would have died of tetanus, or something like it. Now Larrey says, that " in the examination of the bodies of persons dead of tetanus," he " found the pharynx and œsophagus much contracted, and their internal membrane red, inflamed, and covered with a viscid reddish mucus." The rationale then of the treatment in the above cases is, first, to reduce the irritability and tone of the parts exterior to the stomach, and then to brace up the stomach, and restore it to the exercise of its functions. Although distention of the stomach is the primary cause of the disease, it is not to be understood that this condition of the stomach remains throughout the disease the solitary and unassisted cause of all the symptoms. This condition of the stomach forbids sleep, so necessary to the animal functions, and lays a restraint upon all the secretions and excretions in the body. Thus the original cause soon spreads from the stomach over all the vital organs. It is by this very spreading and gradually involving other organs in the disease, and at the same time locking them up so that they cannot assist with their critical discharges, that tetanus, as it advances, gathers so much strength, and, when unassisted, proceeds so rapidly to a fatal conclusion?

*Glasgow, Sept.* 2, 1816. *Annals of Philosophy.*

